# Supplementary material for: A Likelihood Approach for Real-Time Calibration of Stochastic Compartmental Epidemic Models
Source: PLoS Comput Biol. 2017 Jan 17;13(1):e1005257. doi: 10.1371/journal.pcbi.1005257 (PMC5240920; doi:10.1371/journal.pcbi.1005257)
Supplement: S1 File — (TAR.GZ) [file pcbi.1005257.s014.tar.gz › HSPH_Online-SI-Revision/IPoi09/n1-mild/n1-mild_BENCH2_table0.pdf]

1	newly 3 weeks spec	14.	52.5424	{31., 76.}	277.482	0.557364	newly 3 weeks cumul	75.	168.	{125., 217.}	126.486	0.349528	newly 5 weeks spec	14.	40.2034	{21., 59.}	188.862	0.431937	newly 5 weeks cumul	112.	254.847	{177., 331.}	129.964	0.35518	newly 55 weeks cumul	214.	467.	{274., 716.}	119.222	0.317549
2	newly 3 weeks spec	8.	16.4286	{7., 34.}	116.964	0.296505	newly 3 weeks cumul	39.	62.3214	{28., 116.}	75.0916	0.224536	newly 5 weeks spec	6.	10.2679	{3., 21.}	95.5357	0.287243	newly 5 weeks cumul	52.	85.8214	{36., 161.}	79.8077	0.228839	newly 51 weeks cumul	74.	121.143	{42., 240.}	83.6873	0.244799
3	newly 3 weeks spec	19.	9.53125	{0., 29.}	74.8355	1.17227	newly 3 weeks cumul	66.	46.9063	{1., 137.}	80.6345	0.852022	newly 5 weeks spec	13.	1.9375	{0., 7.}	86.5385	1.55657	newly 5 weeks cumul	88.	52.9375	{1., 151.}	75.7102	0.91179	newly 60 weeks cumul	152.	55.5	{1., 151.}	68.5033	1.04531
4	newly 3 weeks spec	7.	27.3864	{11., 49.}	295.779	0.550397	newly 3 weeks cumul	27.	90.8182	{44., 139.}	238.552	0.494414	newly 5 weeks spec	12.	21.9545	{7., 44.}	105.682	0.315108	newly 5 weeks cumul	48.	136.818	{58., 211.}	188.258	0.423146	newly 54 weeks cumul	336.	269.795	{104., 529.}	42.8233	0.25208
5	newly 3 weeks spec	21.	59.7246	{37., 84.}	185.645	0.436709	newly 3 weeks cumul	87.	200.609	{143., 256.}	131.618	0.35409	newly 5 weeks spec	13.	42.5362	{19., 71.}	229.431	0.478581	newly 5 weeks cumul	118.	294.043	{194., 395.}	150.221	0.383695	newly 56 weeks cumul	175.	455.449	{271., 697.}	161.797	0.393963
6	newly 3 weeks spec	25.	70.1471	{34., 96.}	185.176	0.450299	newly 3 weeks cumul	80.	181.824	{68., 263.}	136.397	0.371417	newly 5 weeks spec	28.	72.4559	{51., 94.}	162.027	0.419771	newly 5 weeks cumul	143.	327.074	{177., 424.}	133.289	0.367439	newly 60 weeks cumul	316.	753.588	{658., 859.}	138.477	0.374898
7	newly 3 weeks spec	43.	11.6207	{0., 32.}	75.3809	1.05538	newly 3 weeks cumul	128.	52.6897	{10., 136.}	66.7565	0.610497	newly 5 weeks spec	19.	5.24138	{0., 17.}	78.5844	1.28261	newly 5 weeks cumul	180.	65.3448	{10., 192.}	69.7893	0.690721	newly 59 weeks cumul	287.	73.6207	{10., 251.}	75.2854	0.850427
8	newly 3 weeks spec	17.	33.4348	{19., 48.}	96.6752	0.272018	newly 3 weeks cumul	45.	100.761	{55., 138.}	123.913	0.329768	newly 5 weeks spec	19.	30.6304	{16., 45.}	67.6201	0.213159	newly 5 weeks cumul	80.	163.087	{101., 217.}	104.13	0.294801	newly 51 weeks cumul	385.	427.63	{274., 617.}	28.0576	0.1158
9	newly 3 weeks spec	39.	34.0172	{17., 54.}	29.664	0.153758	newly 3 weeks cumul	109.	117.034	{69., 162.}	25.783	0.109903	newly 5 weeks spec	28.	28.069	{10., 49.}	42.2414	0.205055	newly 5 weeks cumul	160.	176.879	{90., 247.}	31.1099	0.130856	newly 50 weeks cumul	206.	304.655	{137., 468.}	61.232	0.199576
10	newly 3 weeks spec	14.	27.6667	{12., 50.}	103.416	0.261832	newly 3 weeks cumul	60.	100.71	{48., 179.}	75.2415	0.217057	newly 5 weeks spec	11.	19.7826	{6., 46.}	98.8142	0.266296	newly 5 weeks cumul	94.	143.406	{65., 268.}	66.4971	0.203018	newly 51 weeks cumul	109.	228.232	{95., 505.}	114.918	0.275113
11	newly 3 weeks spec	16.	30.38	{15., 41.}	96.625	0.295677	newly 3 weeks cumul	57.	90.46	{53., 123.}	65.7895	0.228372	newly 5 weeks spec	6.	27.86	{13., 38.}	367.	0.639834	newly 5 weeks cumul	78.	148.22	{89., 195.}	94.641	0.291036	newly 50 weeks cumul	387.	418.66	{246., 625.}	28.615	0.125282
12	newly 3 weeks spec	18.	24.6977	{9., 41.}	61.7571	0.27647	newly 3 weeks cumul	54.	74.9302	{22., 126.}	64.5134	0.268552	newly 5 weeks spec	20.	23.2558	{9., 36.}	44.8837	0.230925	newly 5 weeks cumul	85.	123.116	{41., 189.}	65.472	0.248868	newly 51 weeks cumul	271.	442.884	{264., 618.}	70.7543	0.227396
13	newly 3 weeks spec	19.	13.8333	{3., 27.}	55.0877	0.35182	newly 3 weeks cumul	55.	58.0333	{20., 111.}	55.4545	0.245459	newly 5 weeks spec	8.	8.16667	{1., 18.}	74.1667	0.461876	newly 5 weeks cumul	79.	76.4167	{25., 146.}	55.0844	0.265586	newly 52 weeks cumul	97.	99.3333	{29., 189.}	58.2818	0.269244
14	newly 3 weeks spec	15.	35.02	{11., 61.}	140.933	0.338869	newly 3 weeks cumul	78.	130.62	{65., 197.}	75.2051	0.227027	newly 5 weeks spec	10.	23.96	{6., 49.}	152.4	0.331401	newly 5 weeks cumul	112.	182.2	{78., 302.}	74.0357	0.223791	newly 56 weeks cumul	131.	255.2	{94., 468.}	105.557	0.274018
15	newly 3 weeks spec	27.	36.8478	{19., 63.}	56.1192	0.192418	newly 3 weeks cumul	78.	134.13	{66., 217.}	79.5987	0.239323	newly 5 weeks spec	19.	24.8478	{6., 46.}	66.0183	0.254588	newly 5 weeks cumul	124.	189.413	{91., 328.}	65.9712	0.21409	newly 56 weeks cumul	280.	281.674	{107., 498.}	44.7283	0.21073
16	newly 3 weeks spec	11.	37.1154	{19., 60.}	239.86	0.498821	newly 3 weeks cumul	35.	124.154	{64., 191.}	258.242	0.53041	newly 5 weeks spec	16.	27.25	{12., 50.}	85.2163	0.257955	newly 5 weeks cumul	59.	184.038	{102., 286.}	214.928	0.473869	newly 54 weeks cumul	273.	347.904	{159., 570.}	50.0352	0.181089
17	newly 3 weeks spec	20.	41.4151	{25., 56.}	114.811	0.362684	newly 3 weeks cumul	50.	134.208	{83., 182.}	174.151	0.432673	newly 5 weeks spec	12.	34.4906	{17., 53.}	196.855	0.491564	newly 5 weeks cumul	78.	208.208	{146., 285.}	172.738	0.43402	newly 57 weeks cumul	269.	413.943	{210., 603.}	64.1229	0.220771
18	newly 3 weeks spec	14.	34.7167	{19., 50.}	150.595	0.377903	newly 3 weeks cumul	53.	111.233	{67., 155.}	114.34	0.322122	newly 5 weeks spec	18.	34.1833	{19., 44.}	96.0185	0.286564	newly 5 weeks cumul	81.	180.233	{114., 241.}	126.091	0.343579	newly 50 weeks cumul	239.	452.417	{287., 657.}	92.894	0.272458
19	newly 3 weeks spec	24.	57.9365	{33., 88.}	141.402	0.357418	newly 3 weeks cumul	86.	204.508	{135., 269.}	137.8	0.361163	newly 5 weeks spec	24.	40.1587	{19., 79.}	78.5714	0.223811	newly 5 weeks cumul	128.	293.778	{175., 409.}	129.514	0.342539	newly 57 weeks cumul	218.	414.937	{210., 669.}	92.8571	0.257379
20	newly 3 weeks spec	15.	51.6508	{35., 68.}	244.339	0.517084	newly 3 weeks cumul	66.	165.778	{119., 211.}	152.525	0.390167	newly 5 weeks spec	18.	45.9365	{26., 67.}	155.203	0.380848	newly 5 weeks cumul	102.	261.587	{182., 330.}	156.769	0.397253	newly 54 weeks cumul	153.	499.429	{294., 682.}	226.424	0.494291
21	newly 3 weeks spec	19.	6.56667	{0., 16.}	76.3158	1.06333	newly 3 weeks cumul	59.	30.4333	{1., 66.}	68.9831	0.72648	newly 5 weeks spec	16.	3.16667	{0., 8.}	88.5417	1.55554	newly 5 weeks cumul	93.	38.2333	{1., 83.}	70.8602	0.810237	newly 54 weeks cumul	145.	44.9	{1., 104.}	76.2529	0.947279
22	newly 3 weeks spec	17.	36.	{17., 57.}	123.529	0.380274	newly 3 weeks cumul	45.	111.196	{46., 165.}	158.693	0.450019	newly 5 weeks spec	12.	29.5294	{14., 49.}	158.17	0.431284	newly 5 weeks cumul	68.	174.314	{93., 272.}	165.975	0.457403	newly 56 weeks cumul	388.	426.667	{203., 671.}	42.8745	0.247064
23	newly 3 weeks spec	18.	9.71429	{0., 35.}	71.6553	0.72319	newly 3 weeks cumul	69.	35.2653	{2., 117.}	68.8258	0.725849	newly 5 weeks spec	27.	6.20408	{0., 18.}	77.7778	1.12686	newly 5 weeks cumul	118.	49.2653	{2., 164.}	69.9412	0.812381	newly 51 weeks cumul	241.	69.2449	{2., 227.}	74.113	0.995628
24	newly 3 weeks spec	25.	39.4576	{21., 57.}	65.0169	0.209575	newly 3 weeks cumul	83.	128.407	{76., 173.}	61.3641	0.204952	newly 5 weeks spec	20.	36.0339	{20., 55.}	83.3898	0.245191	newly 5 weeks cumul	121.	204.068	{136., 274.}	70.7242	0.221997	newly 53 weeks cumul	298.	459.085	{244., 682.}	60.5619	0.193165
25	newly 3 weeks spec	12.	22.0889	{6., 42.}	106.667	0.312821	newly 3 weeks cumul	34.	76.4	{31., 141.}	132.81	0.337417	newly 5 weeks spec	11.	18.4444	{3., 40.}	99.596	0.340001	newly 5 weeks cumul	59.	114.622	{45., 212.}	107.458	0.301373	newly 50 weeks cumul	239.	211.067	{53., 432.}	50.5346	0.300201
26	newly 3 weeks spec	22.	39.0877	{15., 60.}	92.0255	0.315672	newly 3 weeks cumul	61.	131.947	{63., 188.}	125.165	0.371968	newly 5 weeks spec	13.	30.8421	{14., 52.}	145.074	0.390543	newly 5 weeks cumul	91.	195.719	{82., 285.}	121.477	0.353365	newly 53 weeks cumul	122.	344.632	{160., 561.}	187.863	0.451844
27	newly 3 weeks spec	11.	38.0364	{24., 57.}	245.785	0.513247	newly 3 weeks cumul	44.	121.964	{76., 164.}	178.43	0.428355	newly 5 weeks spec	10.	31.7455	{17., 47.}	217.818	0.467529	newly 5 weeks cumul	72.	188.891	{125., 258.}	163.005	0.402912	newly 50 weeks cumul	169.	404.018	{218., 566.}	139.946	0.355287
28	newly 3 weeks spec	15.	37.4464	{16., 57.}	154.405	0.382584	newly 3 weeks cumul	48.	104.161	{42., 162.}	124.368	0.332305	newly 5 weeks spec	16.	42.375	{24., 58.}	167.076	0.403199	newly 5 weeks cumul	77.	186.518	{87., 260.}	146.22	0.371687	newly 56 weeks cumul	293.	598.821	{461., 728.}	104.571	0.303766
29	newly 3 weeks spec	19.	44.3382	{21., 68.}	136.92	0.353284	newly 3 weeks cumul	60.	145.632	{82., 198.}	144.779	0.374451	newly 5 weeks spec	14.	33.0294	{17., 54.}	136.975	0.337803	newly 5 weeks cumul	96.	218.868	{115., 304.}	129.672	0.342547	newly 53 weeks cumul	143.	382.147	{179., 602.}	167.236	0.390082
30	newly 3 weeks spec	28.	40.5192	{16., 69.}	62.9808	0.212922	newly 3 weeks cumul	82.	150.538	{76., 230.}	88.4615	0.253198	newly 5 weeks spec	11.	24.0385	{7., 44.}	133.217	0.328956	newly 5 weeks cumul	114.	206.596	{107., 338.}	87.7699	0.252011	newly 57 weeks cumul	214.	283.481	{118., 507.}	55.4188	0.201002
31	newly 3 weeks spec	34.	55.875	{34., 83.}	66.5441	0.203549	newly 3 weeks cumul	96.	189.903	{125., 260.}	98.7413	0.284295	newly 5 weeks spec	13.	43.2778	{20., 73.}	233.547	0.478103	newly 5 weeks cumul	127.	283.653	{170., 391.}	123.349	0.330071	newly 55 weeks cumul	143.	455.931	{258., 729.}	218.833	0.472857
32	newly 3 weeks spec	11.	31.6458	{19., 45.}	188.447	0.430861	newly 3 weeks cumul	53.	99.2708	{49., 143.}	92.8066	0.270132	newly 5 weeks spec	12.	30.2917	{16., 45.}	154.167	0.380322	newly 5 weeks cumul	70.	160.729	{92., 220.}	130.268	0.340861	newly 53 weeks cumul	339.	449.583	{267., 642.}	43.2276	0.157142
33	newly 3 weeks spec	38.	52.8033	{35., 70.}	46.3762	0.164382	newly 3 weeks cumul	103.	169.066	{119., 214.}	69.0116	0.228341	newly 5 weeks spec	18.	43.1475	{23., 60.}	140.255	0.355041	newly 5 weeks cumul	138.	260.607	{188., 331.}	91.934	0.278199	newly 54 weeks cumul	205.	490.836	{304., 653.}	139.432	0.359792
34	newly 3 weeks spec	13.	17.2069	{2., 45.}	88.0637	0.423536	newly 3 weeks cumul	28.	60.5172	{9., 123.}	142.241	0.401335	newly 5 weeks spec	11.	12.6552	{0., 37.}	88.4013	0.567694	newly 5 weeks cumul	49.	87.4483	{13., 190.}	110.978	0.364888	newly 53 weeks cumul	176.	163.241	{16., 472.}	72.0219	0.446112
35	newly 3 weeks spec	36.	56.3387	{37., 76.}	60.4391	0.1974	newly 3 weeks cumul	89.	180.226	{131., 226.}	104.023	0.303035	newly 5 weeks spec	34.	48.5484	{32., 63.}	48.8615	0.165492	newly 5 weeks cumul	162.	280.484	{214., 345.}	75.01	0.236116	newly 55 weeks cumul	281.	521.032	{322., 673.}	88.0955	0.261844
36	newly 3 weeks spec	29.	67.6901	{44., 87.}	133.9	0.354503	newly 3 weeks cumul	101.	226.366	{158., 270.}	125.659	0.348275	newly 5 weeks spec	18.	47.2254	{27., 66.}	164.554	0.400063	newly 5 weeks cumul	133.	331.592	{223., 416.}	149.677	0.387662	newly 57 weeks cumul	143.	513.859	{333., 688.}	259.342	0.539001
37	newly 3 weeks spec	16.	27.902	{15., 42.}	82.7206	0.248285	newly 3 weeks cumul	67.	98.902	{55., 147.}	59.1454	0.208582	newly 5 weeks spec	19.	21.1176	{7., 42.}	56.1404	0.25541	newly 5 weeks cumul	103.	144.725	{79., 217.}	56.6914	0.201864	newly 53 weeks cumul	179.	266.706	{104., 494.}	70.0515	0.224035
38	newly 3 weeks spec	31.	51.8235	{28., 79.}	74.0987	0.228396	newly 3 weeks cumul	127.	185.015	{120., 250.}	51.8643	0.179058	newly 5 weeks spec	22.	33.5588	{13., 58.}	71.2567	0.227632	newly 5 weeks cumul	168.	260.206	{150., 366.}	61.5721	0.202415	newly 57 weeks cumul	211.	368.676	{192., 585.}	81.4051	0.241488
39	newly 3 weeks spec	16.	27.9038	{10., 55.}	92.4279	0.257947	newly 3 weeks cumul	52.	102.558	{43., 188.}	106.25	0.27767	newly 5 weeks spec	14.	17.7692	{3., 41.}	88.4615	0.361877	newly 5 weeks cumul	79.	143.712	{58., 282.}	96.4216	0.261859	newly 55 weeks cumul	143.	201.346	{71., 385.}	75.7396	0.253538
40	newly 3 weeks spec	28.	43.5893	{16., 70.}	74.8087	0.249071	newly 3 weeks cumul	115.	157.607	{77., 222.}	52.236	0.188188	newly 5 weeks spec	12.	28.0714	{7., 56.}	153.571	0.359962	newly 5 weeks cumul	156.	220.161	{92., 333.}	58.2532	0.203519	newly 58 weeks cumul	187.	302.196	{113., 537.}	78.772	0.240883
41	newly 3 weeks spec	19.	54.2245	{20., 95.}	191.407	0.425497	newly 3 weeks cumul	73.	192.143	{83., 269.}	166.956	0.4012	newly 5 weeks spec	10.	32.8163	{8., 78.}	238.367	0.469317	newly 5 weeks cumul	107.	265.531	{132., 398.}	153.347	0.381761	newly 59 weeks cumul	145.	358.102	{140., 587.}	154.201	0.37533
42	newly 3 weeks spec	12.	79.4366	{57., 100.}	561.972	0.81027	newly 3 weeks cumul	104.	245.521	{205., 295.}	137.297	0.368636	newly 5 weeks spec	0.1	61.5634	{42., 86.}	61463.4	2.77299	newly 5 weeks cumul	106.	378.718	{322., 447.}	257.281	0.547416	newly 58 weeks cumul	106.	625.211	{462., 772.}	489.822	0.762343
43	newly 3 weeks spec	28.	45.3529	{18., 85.}	79.5168	0.23913	newly 3 weeks cumul	117.	196.662	{98., 329.}	76.7345	0.228184	newly 5 weeks spec	11.	19.5147	{5., 44.}	106.016	0.297261	newly 5 weeks cumul	151.	246.574	{115., 423.}	74.961	0.224862	newly 60 weeks cumul	167.	280.941	{121., 518.}	80.2747	0.232475
44	newly 3 weeks spec	20.	40.6	{23., 61.}	103.	0.28418	newly 3 weeks cumul	78.	129.517	{85., 179.}	67.2436	0.208898	newly 5 weeks spec	6.	28.7333	{14., 44.}	378.889	0.643092	newly 5 weeks cumul	92.	191.6	{117., 268.}	108.261	0.300257	newly 52 weeks cumul	103.	325.517	{185., 478.}	216.036	0.468405
45	newly 3 weeks spec	25.	32.7241	{17., 49.}	45.3793	0.162682	newly 3 weeks cumul	80.	112.534	{65., 163.}	49.1595	0.166704	newly 5 weeks spec	16.	23.8448	{12., 41.}	61.3147	0.190663	newly 5 weeks cumul	114.	164.034	{94., 247.}	52.1476	0.171775	newly 50 weeks cumul	184.	280.069	{134., 448.}	63.943	0.195229
46	newly 3 weeks spec	21.	49.8667	{23., 69.}	138.889	0.35424	newly 3 weeks cumul	65.	158.367	{95., 202.}	144.769	0.376038	newly 5 weeks spec	19.	44.7833	{17., 68.}	141.14	0.351937	newly 5 weeks cumul	90.	248.983	{147., 319.}	176.981	0.424127	newly 57 weeks cumul	370.	447.867	{209., 645.}	41.5135	0.165114
47	newly 3 weeks spec	10.	29.1364	{6., 51.}	209.545	0.487335	newly 3 weeks cumul	30.	85.4318	{19., 152.}	197.955	0.445692	newly 5 weeks spec	5.	31.4318	{10., 55.}	535.	0.765632	newly 5 weeks cumul	45.	147.955	{45., 255.}	238.384	0.497257	newly 55 weeks cumul	300.	496.932	{332., 676.}	74.4318	0.287488
48	newly 3 weeks spec	18.	34.7143	{17., 57.}	97.619	0.269964	newly 3 weeks cumul	78.	117.061	{65., 166.}	56.9335	0.18666	newly 5 weeks spec	4.	29.0612	{12., 50.}	626.531	0.794464	newly 5 weeks cumul	93.	177.694	{92., 269.}	93.9653	0.266293	newly 50 weeks cumul	172.	318.898	{137., 517.}	92.786	0.261592
49	newly 3 weeks spec	6.	36.3556	{19., 57.}	512.593	0.754031	newly 3 weeks cumul	35.	110.8	{65., 162.}	224.952	0.506377	newly 5 weeks spec	12.	33.	{9., 54.}	186.852	0.444401	newly 5 weeks cumul	58.	179.044	{105., 259.}	217.51	0.496583	newly 54 weeks cumul	236.	444.356	{251., 588.}	93.7476	0.295367
50	newly 3 weeks spec	20.	39.3158	{19., 59.}	101.667	0.284218	newly 3 weeks cumul	56.	131.789	{75., 193.}	135.714	0.349109	newly 5 weeks spec	23.	29.3509	{14., 45.}	47.4447	0.179644	newly 5 weeks cumul	95.	196.316	{110., 288.}	108.827	0.299921	newly 51 weeks cumul	220.	326.877	{168., 507.}	61.2919	0.203242
